# Accessibility to palliative care services in Colombia: an analysis of geographic disparities

**DOI:** 10.1186/s12889-024-19132-2

**Published:** 2024-06-21

**Authors:** Miguel Antonio Sánchez-Cárdenas, Marta Ximena León, Luisa Fernanda Rodríguez-Campos, Lina María Vargas-Escobar, Laura Cabezas, Juan Pablo Tamayo-Díaz, Angela Cañon Piñeros, Nidia Mantilla-Manosalva, Genny Paola Fuentes-Bermudez

**Affiliations:** 1 Universidad El Bosque, Bogotá, DC Colombia; 2 School of Medicine, Universidad de La Sabana, Chía, Colombia; 3 Department of Collective Health, Universidad Nacional de Colombia, Bogota, Colombia

**Keywords:** Palliative Care, Colombia, Health services accessibility, Healthcare disparities, Voronoi diagram, Palliative care services

## Abstract

**Objectives:**

Due to the increase in the prevalence of non-communicable diseases and the Colombian demographic transition, the necessity of palliative care has arisen. This study used accessibility and coverage indicators to measure the geographic barriers to palliative care.

**Methods:**

Population-based observational study focused on urban areas and adult population from Colombia, which uses three measurements of geographic accessibility to services: a) density of palliative care services per 100,000 inhabitants, b) analysis of geographic distribution by territorial nodes of the country, and c) spatial analysis of palliative care services using Voronoi diagrams. ArcGIS Pro software was used to map services’ locations and identify geographic disparities.

**Results:**

A total of 504 palliative care services were identified, of which 77% were primary health care services. The density of palliative care services in Colombia is 1.8 primary care services per 100,000 inhabitants and 0.4 specialized services per 100,000 inhabitants. The average palliative care coverage is 41%, two regions of the country have a coverage below 30%. Twenty-eight percent of the services provide care for a population greater than 50,000 inhabitants within their coverage area, exceeding the acceptable limit by international standards.

**Conclusions:**

Palliative care services are concentrated in three main regions (Bogotá D.C., the Center, and the Caribbean) and are limited in the Orinoquia and Amazonia nodes. Density of specialized palliative care services is extremely low and there are regions without palliative services for adults with palliative needs.

## Background

Due to the high prevalence of diseases such as cardiovascular diseases, cancer, chronic respiratory diseases, acquired immunodeficiency syndrome (AIDS), and diabetes, among others, demand for health services that provide end-of-life care has grown among health systems worldwide [1, 2]. Furthermore, the demographic transition, mortality increase, and the fact that there are more than 8 million children with complex diseases every year confirm the present and future nature of the public health emergency caused by chronic non-communicable diseases [1, 3, 4]. About 61 million adults and children worldwide unnecessarily experience health-related suffering that palliative care can address and treat [3]. A palliative care is a intervention that improves the QoL of patients with cancer [5], reduced hospital emergency admissions [6], hospital bed days, hospital costs and increased home deaths and increased regional palliative care access [7, 8]. Additional improve symptom burden [9, 10], family and caregivers well-being and reduce secondary service utilization [11].

Different analyses have identified determining factors for the development of palliative care in national health systems, among which are the availability of specialized palliative care services and their geographic distribution within a country [12, 13]. The adequate provision of health services depends on several factors, and the geospatial location is a relevant factor because it determines access to health services for populations in certain territories [14]. In areas where the population is geographically more dispersed, access to specialized palliative care interventions is limited [15]. Palliative care services are concentrated in urban areas where referral hospitals have the capacity to provide care for a high volume of patients from surrounding regions that have lower specialized care capacity; such concentration of palliative care services causes migration of patients and their families who come to cities in search of disease treatment or an end of life without suffering [16]. Geographic distribution of populations and health services can represent barriers to treatment access for relieving end-of-life severe suffering [17, 18]. For the World Health Organization (WHO), geographic accessibility is measured by the time a patient must travel to a health facility, and a maximum time of two hours is considered standard; the percentage of the population living within 5 km (3.1 miles) of a health facility is a measurement also used, especially for access to surgical or emergency services [19]. In palliative care, a commonly used indicator is the density of services per 100,000 inhabitants; however, this indicator does not consider the distribution of the services in the national territory, so the identification of more objective and simple mechanisms that allow the use of official databases on health care institutions to assess density and distribution is necessary [20].

In Latin America, access to palliative care is inequitable for limited palliative care services [21], low availability and barriers of access to opioid medicines [22], low proportion of training programs for professionals in palliative care [23], geographic access [24], lack of funding and misconceptions about palliative care [25]. In Colombia, access barriers related to the health system’s structural characteristics and regions' geography have been identified [26]. Despite significant advances in the provision of services in the territory and public health policies to ensure adequate availability of palliative care and uphold the right to health, there is still evidence of coverage problems in many areas of the national territory, especially in places other than the country’s large cities [27].

In the literature available on geographic and demographic health care analysis, information from official sources and government databases is extensively used [28] as well as statistical and cartographic methods for processing the available data on palliative care at the international level [29–31]. However, there is little evidence locally of the use of available data for carrying out population-based studies. This study evaluates the geographic density and distribution of palliative care services, estimating the coverage of palliative care services for the urban adult population residing in Colombia to determine the accessibility to these services and establish a point of comparison with the region and the world.

## Methods

### Type and field of study

Population-based observational study [32] conducted in Colombia (South America) a country with multi-diverse geography and population [33]. It is divided into 32 departments, 1123 municipalities, and a capital district (Bogotá, D.C.) [33]. The total estimated population is 48,258,494 people, 77.5% of whom are over 15 years of age [34].

### Design

A descriptive and geographic analysis of density, coverage, and distribution of palliative care services for adults in urban areas of the Colombian territory was developed. The Colombian Ministry of Health and Social Protection’s records in 2020 [35] were used as an official source of information on the number and geographic location of palliative care services. Information on the urban population aged over 20 years was obtained from the 2018 National Population and Housing Census [36]. Considering that the identified palliative care services are not offered in rural areas, nor do they provide care for minor children, the population with these characteristics was excluded from the study.

### Density of palliative care services by population

Specialized and primary palliative care services were identified in each region. The palliative care services rate per 100,000 inhabitants was calculated to identify the regions with a suboptimal proportion of services available to meet the health demand. In addition, the total number of inhabitants aged over 20 years residing in the observed regions was also used for this calculus [36].

### Geographic distribution of palliative care services

For assessing the geographic distribution of palliative care services in Colombia, each palliative care service’s address was geocoded using ArcGIS Pro and was verified using the data available in OpenStreetMap (OSM) collaborative project [37, 38]. Later, zones of influence for each geographical location of the services were established, so a region comprised the nearest neighbors of a service in the geographical space, as defined by the method of Voronoi diagram [39]. The Voronoi diagrams are a most helpful tool to conduct observational studies with descriptive analyses in health care because they facilitate clustering elements together in a geospatial setting. Then, georeferencing of the population aged over 20 years and distributed by blocks in urban areas of Colombia was performed [36]. The number of inhabitants residing in each polygon created to represent areas of influence of specialized palliative care services and the geographic distribution of the services according to population and regions’ coverage needs were identified.

### Data processing

#### Geographic analysis

The model proposed by León et al. was used for the geographical analysis [40]. This model consists of grouping the 33 regions of the country into seven clusters or nodes (see Table 1), which were established in a previous study about the development of palliative care in Colombia. Each node groups territories according to sociodemographic characteristics [40]. The total number of palliative care services per node and the regions where they are offered were identified.

**Table 1:** Territorial division by nodes

Node	Regions
Amazonia	Putumayo, Amazonas, Caquetá, Guaviare, Vaupés, and Guainía
Orinoquia	Meta, Vichada, Casanare, Arauca, and Cundinamarca
Northeast	Boyacá, Santander, Norte de Santander, and Cesar
Pacific	Nariño, Cauca, Valle del Cauca, and Chocó
Center	Antioquia, Caldas, Quindío, Risaralda, Tolima, and Huila
Caribbean	La Guajira, Magdalena, Atlántico, Bolívar, Sucre, Córdoba, and San Andrés
Bogotá D.C	Bogotá

#### Density of services

The services rate per 100,000 population was analyzed using measures of central tendency. The results are described for each country’s geographical node, establishing a mean of this indicator in each region. Regions with no palliative care services were excluded from the analysis.

#### Spatial distribution

The spatial distribution of palliative care services was analyzed using Voronoi diagrams [24]. This method of cartographic analysis allowed geographical space to be divided according to palliative care services, so that each service was assigned a region that includes all that is closer to him compared to others. services in an established geographic space. In addition, this procedure made it possible to identify areas of the territory defined as coverage areas [39, 41]. Even though some inhabitants may not have access to certain services available in their territory due to administrative issues (e.g., insurance status), this analysis offers a perspective on geographic accessibility to palliative care services and estimates the population currently affected by access barriers.

## Results

### Geographical analysis

A total of 504 palliative care services were identified, of which 77% (*n* = 393) correspond to primary healthcare services that report palliative care services to the Ministry of Health and Social Protection. Table 2 shows the distribution of the population aged over 20 years residing in urban areas and the palliative care services identified. The Caribbean node is the region with the highest number of primary palliative care services (*n* = 108), followed by the Central node (*n* = 85), and Bogotá D.C. (*n* = 74). Specialized palliative care services are mainly located in the Caribbean node (*n* = 31), Bogotá D.C. (*n* = 25), and the Central node (*n* = 22). Due to their geographic location, the Orinoquia and Amazonia nodes have a smaller population living in urban areas and the lowest absolute records of primary and specialized palliative care services. Palliative care services were not identified in six regions (Amazonas, Guainía, Putumayo, Vaupés, Vichada, and Archipelago of San Andrés, Providencia, and Santa Catalina); most of them belong to the Amazonia node (see Table 2).

**Table 2:** Primary and specialized palliative care services by node

Node	Regions	Resident population in the region (%)	Primary palliative care services	Specialized palliative care services
Bogotá	Bogotá, D.C	24.2	74	25
	**Subtotal**	**24.2**	**74**	**25**
Center	Antioquia	13.7	51	9
	Caldas	2.2	9	2
	Quindío	1.4	4	3
	Risaralda	1.8	9	5
	Tolima	1.6	5	2
	Huila	1.3	7	1
	**Subtotal**	**22.0**	**85**	**22**
Caribbean	La Guajira	0.7	5	1
	Magdalena	1.6	13	4
	Atlántico	6.1	37	8
	Bolívar	6.9	26	13
	Sucre	2.0	10	2
	Córdoba	2.1	17	3
	**Subtotal**	**19.3**	**108**	**31**
Pacific	Nariño	2.2	9	4
	Valle del Cauca	10.1	34	9
	Cauca	1.1	6	3
	Chocó	0.5	2	0
	**Subtotal**	**13.8**	**51**	**16**
Northeast	Boyacá	1.8	9	1
	Santander	5.5	22	9
	Norte de Santander	3.3	8	1
	Cesar	1.8	13	3
	**Subtotal**	**12.4**	**52**	**14**
Orinoquia	Meta	1.9	6	2
	Casanare	0.7	1	0
	Arauca	0.3	1	0
	Cundinamarca	4.1	12	1
	**Subtotal**	**7.0**	**20**	**3**
Amazonia	Caquetá	1.0	2	0
	Guaviare	0.2	1	0
	**Subtotal**	**1.2**	**3**	**0**
**Overall results**		100	393	111

### Density of services by population

The density of primary palliative care services in Colombia reaches a rate of 1.82 services per 100,000 inhabitants, with a mean of 14 ± 10 services. The Caribbean node showed a rate of 2.62 non-specialized palliative care services outside the rate recommended by international standards (2 services per 100,000 inhabitants) [13].

**Fig. 1:**
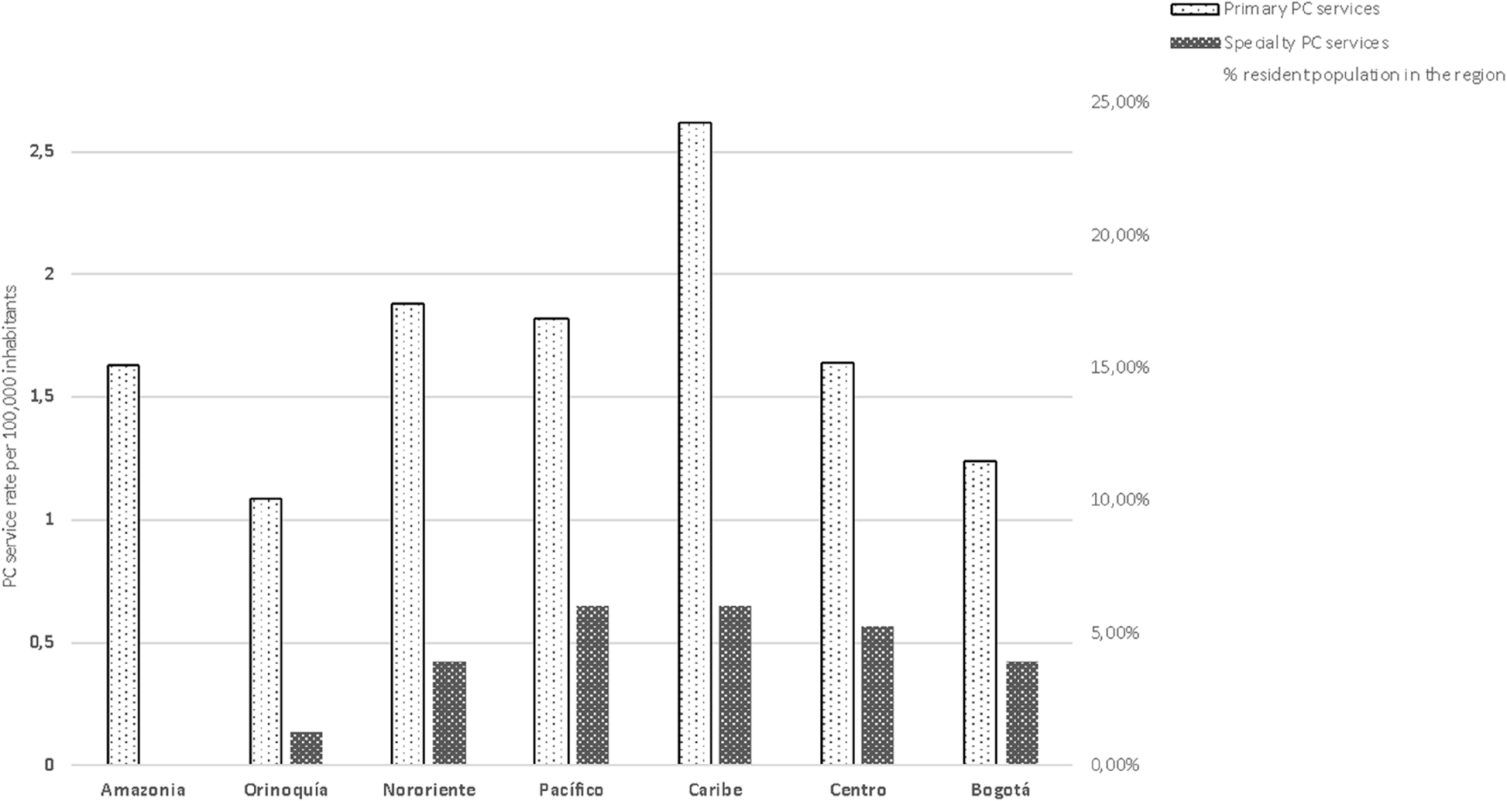
Density of palliative care services per 100,000 inhabitants

The three regions with the highest population concentration in Colombia (Bogotá D.C., Antioquia, and Valle del Cauca) have a service rate of 1.4 per 100,000 inhabitants. Despite the small number of services identified (*n* = 6) in the regions with the fewest inhabitants (2.3% of the population), these regions have a service rate of 1.6 (see Fig. 1). The mean rate of specialized services is 0.47 per 100,000 inhabitants, lower than one service per 100,000 inhabitants, which is the rate internationally recommended. The Caribbean and Pacific nodes have the highest rate of specialized palliative care services (0.65 per 100,000 inhabitants). In contrast to these nodes, the Orinoquia and Amazon nodes have the lowest rate of specialized palliative care services (0.13 and 0 services per 100,000 inhabitants, respectively).

The density rate of specialized palliative care services per 100,000 inhabitants reaches similar values in Bogotá D.C. and Northeastern nodes (0.4 services), and higher in the Central, Caribbean, and Pacific nodes despite the lower supply of specialized services per number of inhabitants in these regions have (see Fig. 1).

### Spatial distribution

**Fig. 2:**
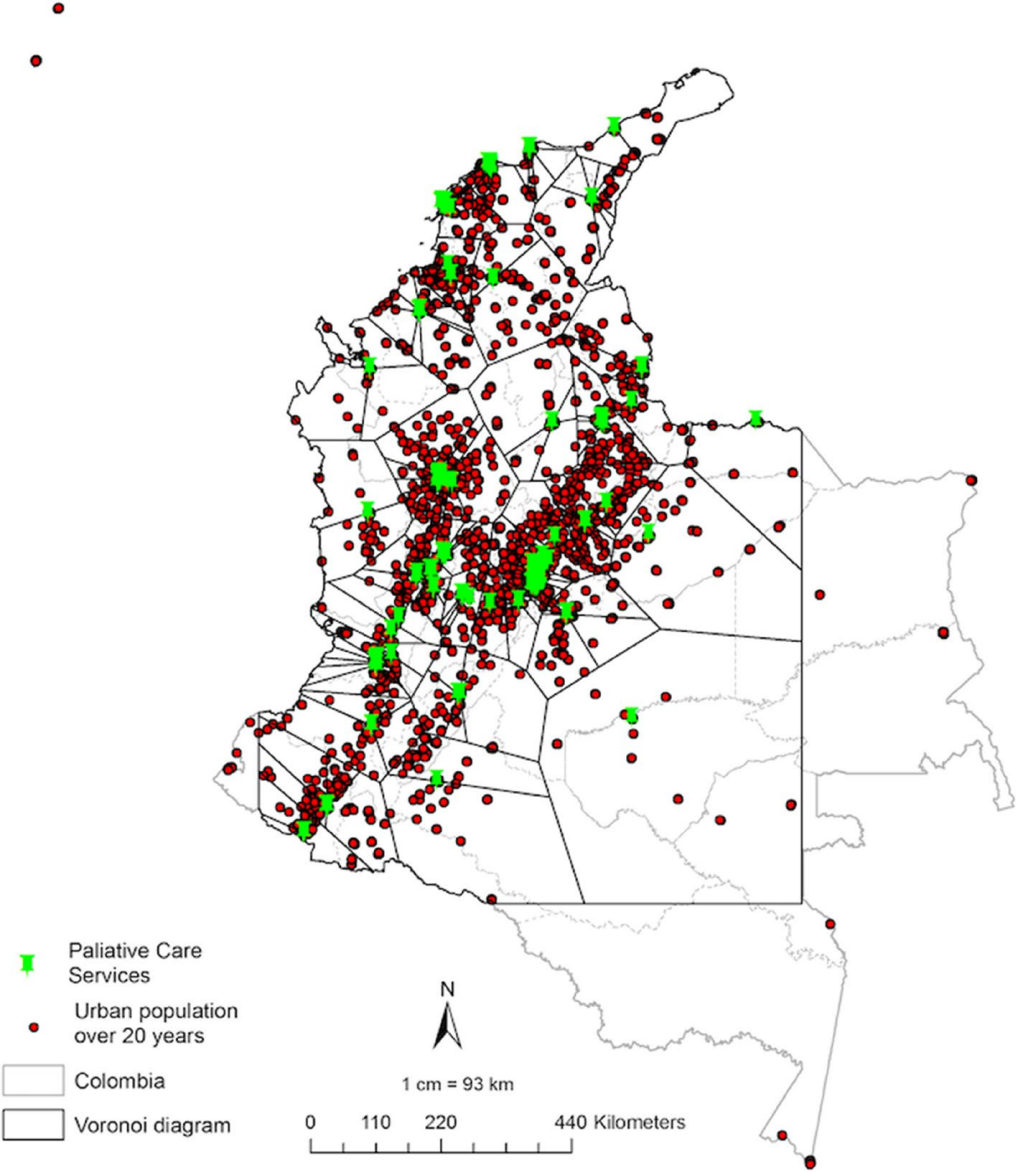
Voronoi diagram of palliative care services and urban population aged over 20 years in Colombia

There are uncovered areas that comprise jungle or island regions without palliative care services; this is the case of the territories of the Amazonia node (Amazonas, Guainía), the Orinoquia node (Vichada), and the Caribbean node (San Andrés). Palliative care services in the Orinoquia and Amazonia nodes are few, so the coverage areas represented by the Voronoi polygons cover large areas of the territory, increasing the population reliant on the nearest available services (see Fig. 2).

An average of 49,150 people per service was identified in the polygons evaluated, and it was higher than the international standard in Bogotá D.C. (*n* = 64,188) and the Centro (*n* = 51,173) nodes. Due to its low service concentration, the Amazonia node has the highest number of people within a Voronoi polygon (*n* = 72,061). Twenty-eight percent of the total services have a population larger than 50,000 inhabitants within their coverage area, mostly in the Amazonia (67%) and Orinoquia (57%) nodes.

Colombia’s services cover 41% of the population. The nodes with the widest coverage are the Bogotá D.C. node (79%), the Center node (46%), the Northeast node (46%), and the Caribbean node (38%). Conversely, Orinoquia and Amazonia nodes have the lowest coverage (29% and 18%, respectively).

## Discussion

Colombia is an upper-middle-income country, and it belongs to the 3b category according to the Mapping Levels of Palliative Care Development in 2017, which means it has a generalized palliative care provision in the territory [42]. However, according to the study conducted by Sánchez-Cárdenas et al., all Colombian regions have a suboptimal supply of palliative care services because no region surpasses the indicator of density of services per 100,000 inhabitants [20, 24]. When the density of services is examined, there is a shortage of palliative care services in the Amazonia and Orinoquia regions located in the south and east of the country, respectively. This supply restriction in jungle and rural regions has caused an increase in the migration of people to places where palliative care services are available [27].

The Caribbean node has the highest density indicators per 100,000 inhabitants, both in primary and specialized palliative care services. However, in nodes where a large proportion of services are concentrated, such as Bogotá D.C., Center, and Pacific nodes, a suboptimal density is observed considering the high demand and the large urban population. It is worth pointing out here that the population proportion living in each region should be considered during the application and analysis of international indicators, such as services density per 100,000 inhabitants, to avoid misinterpreting the actual access to palliative care and so, producing a bias. For this reason, the indicator traditionally used has its drawbacks when subnational territories are assessed, and it can lead to confusing conclusions if they are not carefully evaluated. Therefore, our study’s proposal to use databases from palliative care services to assess density and distribution is relevant in this case.

According to León et al., the Colombian regions with the highest number of deaths from diseases that might require palliative care in 2017 were Bogotá D.C., Atlántico, Risaralda, Santander, and Antioquia. On the other hand, Amazonas, Guainía, Vaupés, Putumayo, and Vichada, report a lower number of deaths than other regions of the country [24]. This fact shows the difference in palliative care service needs of the populations living in different Colombian regions. When the findings on population coverage and geographic distribution of palliative care services are examined, a concentration pattern can be seen in the central, western, and northern regions of the country, especially in Bogotá D.C., Antioquia, and Valle del Cauca. There is a greater concentration of urban population in these regions and a higher prevalence of people with serious illnesses that may require palliative care.

This study provides updated information on the requirements for adults in each region in Colombia to access palliative care services and identifies urban areas where geographic access barriers exist. This work was carried out by contrasting service provision indicators and population coverage, aiming to guide future policymaking to fill the gap in accessibility to palliative care services in the country. The limitation of the present study relates to the use of international palliative care coverage indicators created in developed countries because these standards have not been adapted for Latin America and, therefore, do not consider the capacity of the services according to the available resources in the region. Future research should identify a standard of services per population more appropriate for the local context and consider aspects such as services typology and the health services’ capacity in the regions within the country.

## Conclusions

This analysis of density and geographic distribution exposes inequities in palliative care provision in different Colombian regions representing an access barrier to treatments for alleviating end-of-life severe suffering. Knowing the available health services in a country to alleviate the suffering caused by advanced diseases can help guide the development of public policies with a positive impact on the population. In addition, work should be done in Latin America to develop a service coverage indicator for low- and middle-income countries.

## Data Availability

Data management and sharing All Colombian Observatory Palliative Care data can be accessed from www.occp.com.co or can be asked from occp@unbosque.edu.co.

## Abbreviations

D.C. in Spanish: Capital District
OSM: Open Street Map
WHO: World Health Organization
AIDS: Acquired Immunodeficiency Syndrome
